# In-silico molecular designs to treat neurologic and ophthalmologic diseases caused by sorbitol excess: engineering the *Agrobacterium vitis* protein

**DOI:** 10.1186/s13104-023-06367-2

**Published:** 2023-07-03

**Authors:** Shonit Nair Sharma, Ashkhan Hojati, Bhargavee Gnanasambandam, Rahul S. Yerrabelli, Joshua Brozek

**Affiliations:** 1 Department of Biological Engineering, Massachusetts Institute of Technology, Cambridge, MA 02139 USA; 2 Brigham and Women’s Hospital, Harvard Medical School, Boston, MA 02115 USA; 3 Carle Illinois College of Medicine, University of Illinois at Urbana-Champaign, Champaign, IL 61801 USA; 4 Carle Foundation Hospital, Urbana, IL 61801 USA

**Keywords:** Biologic, Protein Engineering, PyRosetta, Cataract, Neuropathy

## Abstract

This work presents the design of a new protein based on the adenosine triphosphate-binding cassette (ABC) transporter solute binding protein (SBP) derived from *Agrobacterium vitis*, a gram-negative plant pathogen. The Protein Data Bank in Europe’s dictionary of chemical components was utilized to identify sorbitol and D-allitol. Allitol bound to an ABC transporter SBP was identified in the Research Collaboratory for Structural Bioinformatics Protein Data Bank (RCSB). Wizard Pair Fitting and Sculpting tools in PyMOL were used to replace bound allitol with sorbitol. PackMover Python code was used to induce mutations in the ABC transporter SBP’s binding pocket, and changes in free energy for each protein-sorbitol complex were identified. The results indicate that adding charged side chains forms polar bonds with sorbitol in the binding pocket, thus increasing its stabilization. In theory, the novel protein can be used as a molecular sponge to remove sorbitol from tissue and therefore treat conditions affected by sorbitol dehydrogenase deficiency.

**Supplementary Information:**

The online version contains supplementary material available at 10.1186/s13104-023-06367-2.

## Introduction

**Fig. 1:**
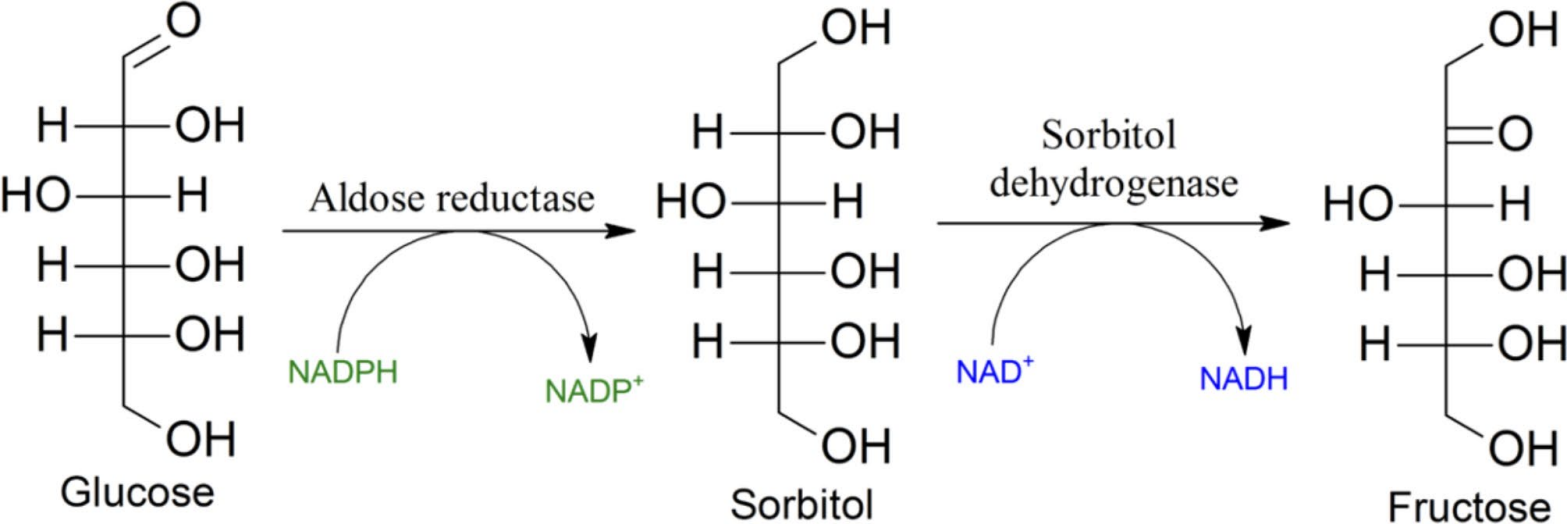
Polyol pathway. The metabolic pathway converts glucose to fructose

The polyol pathway is a biological mechanism utilized by tissues to convert glucose to fructose through converting glucose into an intermediary compound, sorbitol [1]. Sorbitol is the alcohol counterpart of glucose [2]. Its structure effectively traps glucose within a cell, and is converted to fructose via sorbitol dehydrogenase (SORD), as seen in Fig. 1 [3, 4]. Tissues with insufficient sorbitol dehydrogenase can suffer from intracellular sorbitol accumulation, and subsequently osmotic damage. Excess sorbitol may deposit in the eyes leading to cataracts or retinopathy, or damages nerves such as in peripheral neuropathy, similar to the effect of chronic hyperglycemia in diabetes [5–7].

Sorbitol dehydrogenase deficiencies currently affect one in 100,000 individuals and primarily manifest as the most common form of autosomal recessive peripheral neuropathy [8]. In patients with diabetes, excess glucose in the blood significantly increases the production of sorbitol and can lead to cell lesions. In the lens of the eye, diabetic patients can develop cataracts up to 20 years earlier [9] because of osmotic imbalance from excess sorbitol and oxidative damage [10]. These conditions can have drastic effects on quality of life and will require lifestyle changes and long-term management if not addressed in a timely manner.

Nature’s proteins provide an array of engineering possibilities. Through in-silico modeling, proteins can be enhanced (i.e. via mutations), evolving to perform novel functions [11–13]. In-silico molecular design continues to advance, offering improved optimization strategies through machine learning algorithms, peptide modeling, and binding energy analysis [14].

To address the ophthalmic and neurological pathologies associated with sorbitol dehydrogenase deficiency, we propose a protein capable of binding sorbitol with high affinity and specificity. This protein, the adenosine triphosphate-binding cassette (ABC) transporter solute binding protein (SBP), is derived from the microbe *Agrobacterium vitis* (also known as *Allorhizobium vitis*), a Gram-negative, motile, plant pathogen that infects grapevines [11]. The Agrobacterium family has recently been shown to cause certain pathologies like endophthalmitis and pneumonia, delusions from Morgellans disease, and cancer [15]. However, there have been limited findings on *A. vitis* for ophthalmological or neurological pathologies.

The ABC transporter SBP itself has a ‘Venus flytrap’-like mechanism that allows for large conformational changes in its binding domains to allow for the encapsulation of ligands [16]. The structure of the ABC transporter SBP is specific for organic compounds such as allitol or the amino sugars glucosamine and galactosamine [16, 17].

**Fig. 2:**
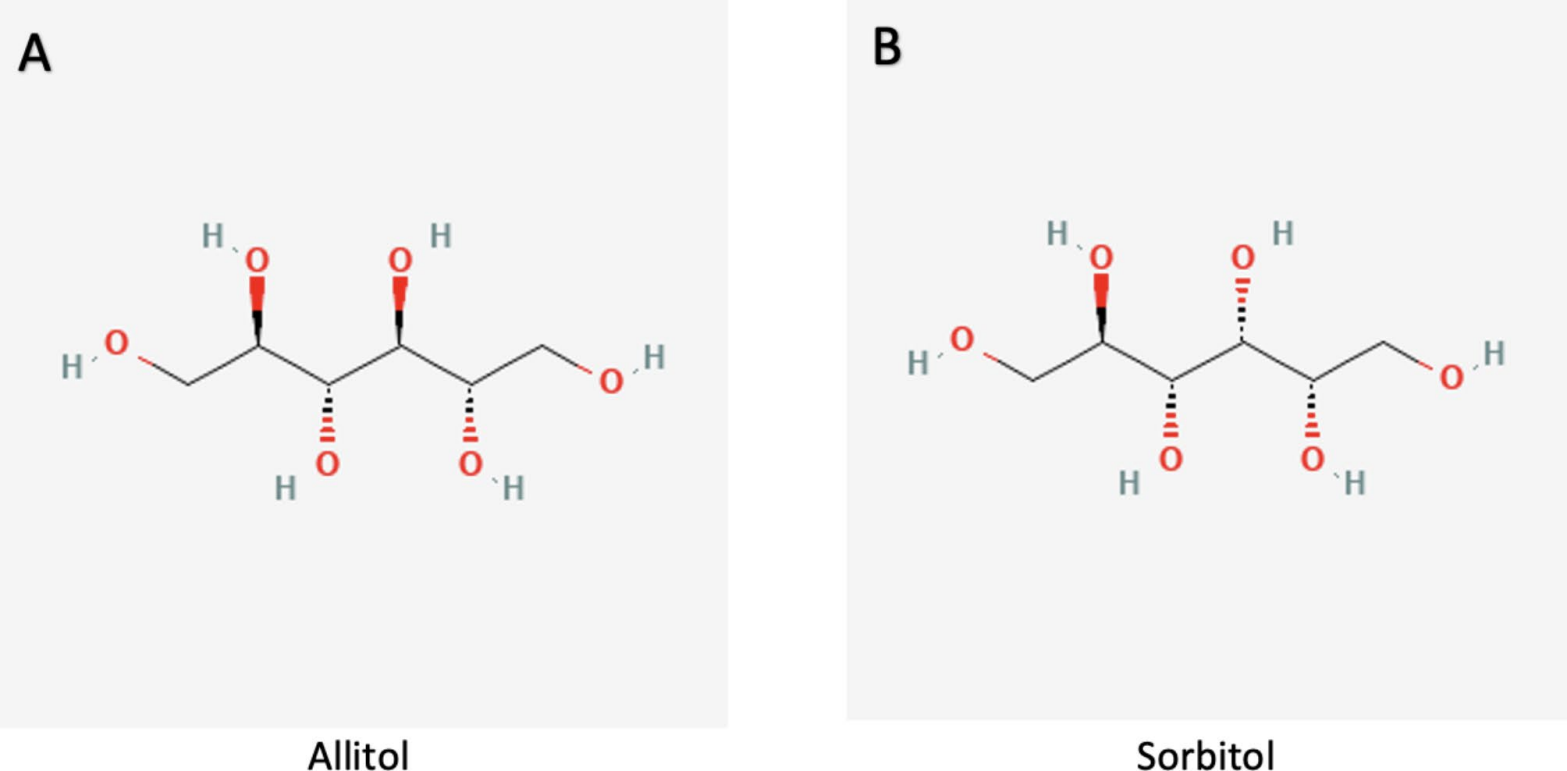
Ligands. (**A**) Allitol is the naturally bound ligand of the ABC transporter SBP. (**B**) Sorbitol, which differs in stereochemistry from allitol only at the third carbon hydroxyl group, is the ligand that the ABC transporter SBP can be engineered for preferential binding

The small molecule allitol (i.e. D-allitol) has a molecular formula of C_6_H_14_O_6_. It is a natural ligand for the ABC transporter SBP. Sorbitol, a structurally similar small molecule, has the same molecular formula as allitol. Both molecules have six-carbon with each carbon attached to a hydroxyl (-OH) group. The difference between allitol and sorbitol is simply the stereochemistry of a single hydroxyl group at the third carbon position, as seen in Fig. 2. The similarity in these two structures suggest that minor modifications to the binding pocket of the ABC transporter SBP can be used as a molecular sponge that preferentially binds sorbitol over allitol. This molecular sponge could decrease excess intracellular sorbitol and limit disease progression in affected individuals. The development of such protein could be utilized as a starting framework for potential candidate medications for the treatment of cataracts, retinopathy, or peripheral neuropathy in individuals suffering from sorbitol dehydrogenase deficiency.

## Methods

### Generating files for use in PyMOL

Utilizing the Protein Data Bank (PDB) in Europe’s Chemical Components in the PDB, we identified that sorbitol, code name of SOR [18], is a related compound of D-allitol, code name of X9X [19]. We then searched the RCSB (Research Collaboratory for Structural Bioinformatics Protein Data Bank 2021) PDB (‘X9X’ 2021, ‘SOR’ 2021) for a protein that contained allitol as a ligand in its structure, protein code name 4WT7 (‘Crystal structure of an ABC transporter solute binding protein (IPR025997) from Agrobacterium Vitis (Avi_5165, Target EFI-511223) with bound allitol’) [17]. We were able to download a ‘.pdb’ file directly for 4WT7. However, this protein was a dimer of two identical monomers. We then deleted one monomer and saved the protein as a new ‘.pdb’ file. As X9X was contained within the structure of 4WT7, we isolated the ligand in the file and saved it as a separate ‘.pdb’ file. Finally, a ‘.sdf’ file for SOR was acquired which was run through an online web service integrated with OpenBabel to convert the file to a ‘.mdl’ file format [20]. This file was converted to a ‘.pdb’ file using the ‘molfile2params’ code.

The above-mentioned ‘.pdb’ files were used in conjunction with PyMOL to calculate energy scores, run experiments, and generate images. A summary of these ‘.pdb’ files is contained in Table 1.

**Table 1:** Summary of ‘.pdb’ files used. Each ‘.pdb’ file with its corresponding nickname that we use in subsequent sections is listed in the table, along with a brief description of the protein and/or ligand the file contains

Ligand/Protein/Complex Nickname	‘.pdb’ File	Details
Protein	protein.pdb	ABC transporter SBP (4WT7) which is downloaded from the PDB as a dimer
Allitol	allitol.pdb	Allitol (X9X) which is isolated from protein.pdb
Sorbitol	sorbitol.pdb	Sorbitol (SOR) which is generated via conversion through OpenBabel and ‘molfile2params’ code
Old Protein	proall.pdb	Protein-allitol complex which is the protein.pdb file but modified to be a monomer
New Protein	prosor.pdb	Protein-sorbitol complex which is essentially proall.pdb plus sorbitol.pdb minus allitol.pdb; the sorbitol molecule is docked in the binding pocket of the protein aligned to the carbon backbone of the allitol molecule before deleting allitol
New Protein after MinMover	minprosor.pdb	Protein-sorbitol complex after being run through the MinMover code
Mutated Protein	finprosor.pdb	Protein-sorbitol complex with selective mutation of 5 residues
New Protein after FastRelax	fastprosor.pdb	Protein-sorbitol complex after being run through the FastRelax code

### Docking ligand to protein in PyMOL

**Fig. 3:**
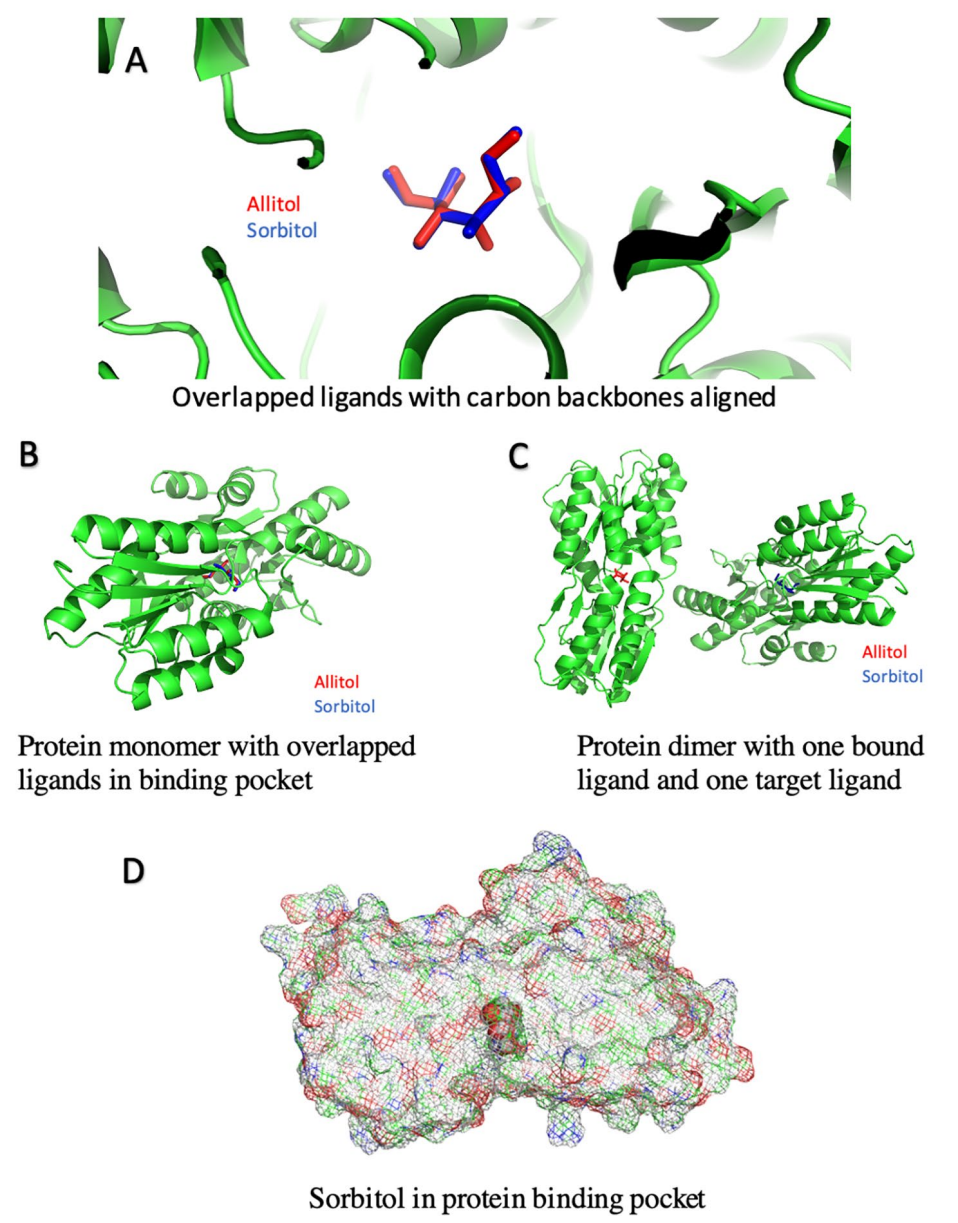
Ligands in the Protein Binding Pocket. **A-C**. The ABC transporter SBP (in green) binds the ligand allitol (in red) in its binding pocket. The ligand sorbitol (in blue) can be docked in the same binding pocket aligned with the six-carbon backbone of allitol. **D.** The ligand sorbitol (solid) visualized inside the binding pocket of the protein (translucent mesh) with atoms specified by color (red = Oxygen, blue = Nitrogen, green = Carbon, white = Hydrogen)

For docking, we first opened protein.pdb in PyMOL and then imported sorbitol.pdb the file into the open PyMOL window [21]. We utilized a combination of the Wizard Pair Fitting and Wizard Sculpting tools in PyMOL to align the carbon backbone of the sorbitol molecule from the sorbitol.pdb file to the carbon backbone of the allitol molecule from the .pdb file (Fig. 3). The Wizard Pair Fitting tool allowed for movement and orientation of the sorbitol molecule while the Wizard Sculpting tool allowed for rotation of bonds to align the hydroxyl groups of sorbitol to those of allitol. This was an iterative process as sometimes using the Wizard Sculpting tool would cause unwanted movement of the sorbitol molecule, so the Wizard Pair Fitting tool was used again to re-align the carbon backbone, and once realigned, identified additional need for the Wizard Sculpting tool. Once the alignment of both the carbon backbone and hydroxyl groups (all but the hydroxyl group of the third carbon, as that differs in stereochemistry) was accomplished, the atoms of the allitol molecule were deleted from the complex. The subsequent protein-sorbitol complex was saved as a new molecule and ‘.pdb’ file.

### Minimizing protein-ligand complex

For minimization, we used the MinMover code to find a local energy minimum of the new protein and the FastRelax code to find a structure with lower energy than the new protein. The results of each of these minimization methods are discussed in the Results section.

### Mutating protein-ligand complex

Given that sorbitol fits in the binding pocket of the protein where allitol once was, we hypothesize that the amino acid side chains stabilizing bound allitol would similarly stabilize much of sorbitol; however, the difference in stereochemistry of the third carbon hydroxyl group between allitol and sorbitol suggests that modification to the protein residues in binding pocket could preferentially bind one ligand over the other. To determine which residues to change, we performed an iterative experiment. We first used PyMOL to identify all residues of the protein within 4 Angstroms of the bound ligand sorbitol. We then used PackMover Python code to change these residues that enhance residue-residue or residue-ligand interactions to further stabilize sorbitol in the binding pocket. To maintain control over design, we generated a protein with only specific amino acids in the binding pocket that was mutated. We did this by running code to change the ‘resfiles’ values of these amino acids from NATRO (no mutation, no repacking) to ALLAA (all amino acids, all repacking) to subsequently change the amino acids. This method generated a protein with all mutated residues in the binding pocket and selectively mutated residues in the binding pocket. We further analyzed the selectively mutated protein.

## Results

### Selective mutation experiment

While in the binding pocket of the ABC transporter SBP, sorbitol has direct polar interactions with five residues in the binding pocket. There are a total of seven bonds created between the ligand and the five amino acid side chains. The details of these interactions are detailed in Table 2. The amino acids in the binding pocket of the ABC transporter SBP also include five side chains that appear to have the potential to participate in polar bonds, which were the target of the selective mutation experiment. Following the experiment, the polar bonds that were created among these residues are listed in Table 3.

**Table 2:** Residues with polar bonds before mutation. The five residues listed create a total of seven polar bonds with the ligand sorbitol when sorbitol is in the binding pocket of the protein prior to mutating any residues in the binding pocket of the protein

	Before Mutation		After Mutation		After FastRelax	
Residue Number	Residue	Number of Polar Bonds	Residue	Number of Polar Bonds	Residue	Number of Polar Bonds
61	HIS	1	HIS	1	HIS	1
89	ARG	1	ARG	1	ARG	2
187	ARG	3	ARG	3	ARG	2
268	ASP	1	ASP	1	ASP	0
290	GLN	1	GLN	1	GLN	1

**Table 3:** Residues with potential for polar bonds. The five residues listed were within four Angstroms of the bound ligand sorbitol and did not create any polar bonds, though were oriented toward the ligand and appeared to have the potential to interact

	Before Mutation		After Mutation		After FastRelax	
Residue Number	Residue	Number of Polar Bonds	Residue	Number of Polar Bonds	Residue	Number of Polar Bonds
63	PHE	0	LYS	0	PHE	0
64	ASP	0	PRO	0	ASP	3
137	ASP	0	TYR	0	ASP	1
181	VAL	0	VAL	0	VAL	0
184	CYS	0	ILE	0	CYS	0

**Fig. 4:**
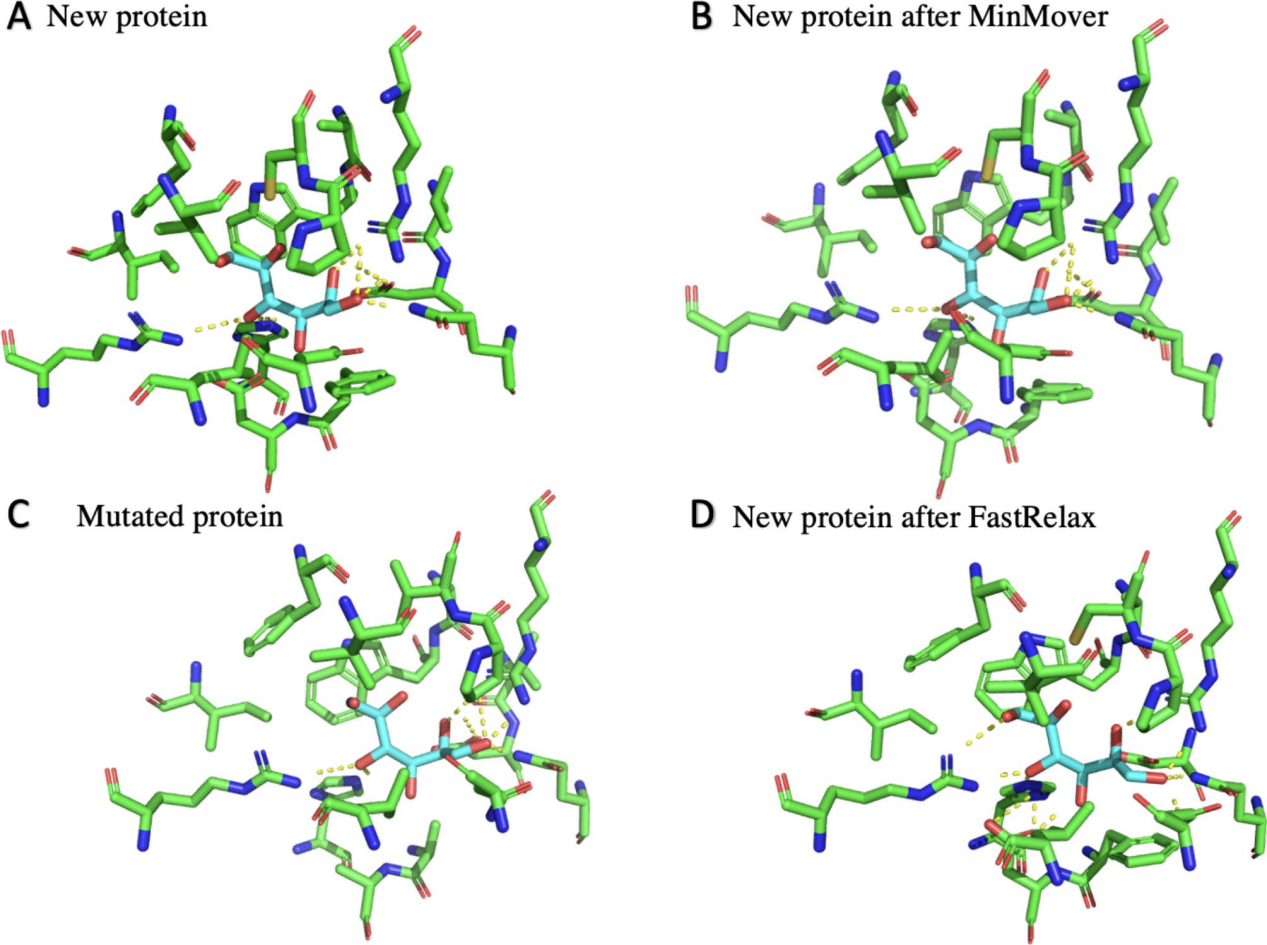
Sorbitol Polar Bonding in Protein Binding Pocket. Sorbitol (cyan) making polar bonds (yellow dashed line) with surrounding binding pocket residues. Highlighted atoms of sorbitol and residues are denoted by their color (Oxygen = red, Nitrogen = blue, Sulfur = yellow, Carbon = green)

For the residues with polar bonds before mutation, once the mutation occurs the number of polar bonds does not change. Additionally, while after FastRelax the number of polar bonds decreases, it is imperative to note that there is an increase in other polar bonds formed among the residues that were identified as ones that had the potential for polar bonds, and thus an overall increase in the number of polar bonds formed (Fig. 4).

**Fig. 5:**
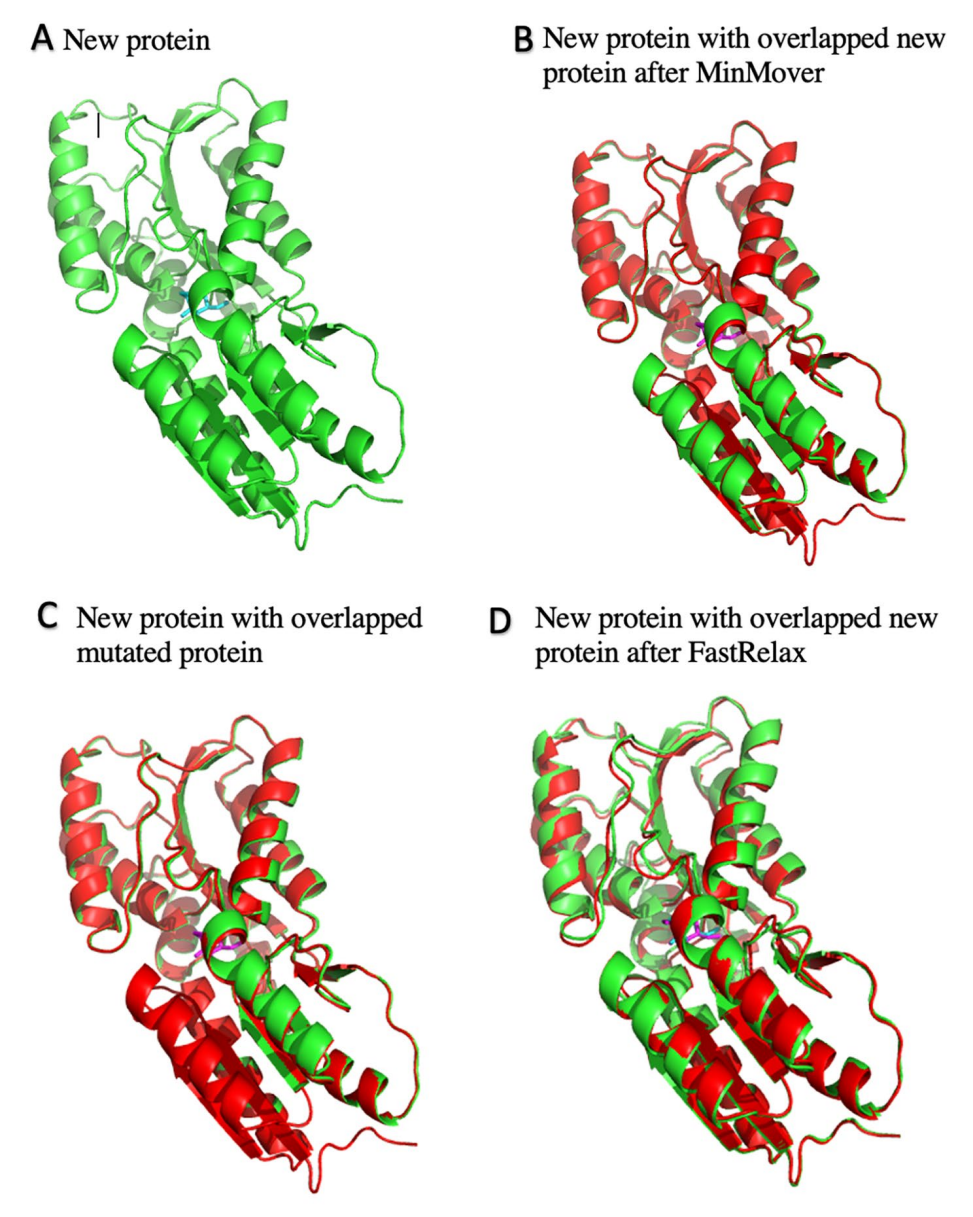
Protein-Sorbitol Complex Structural Changes. (**A**) Sorbitol (cyan) can be seen in the binding pocket of the new protein (green). (**B**) Sorbitol (magenta) can be seen in the binding pocket of the new protein after MinMover (red). (**C**) Sorbitol (magenta) can be seen in the binding pocket of the mutated protein (red). (**D**) Sorbitol (magenta) can be seen in the binding pocket of the new protein after FastRelax (red)

### Free energy changes associated with each complex

Different blocks of Python code were run at each step of the experiment to calculate energy scores for the new protein and subsequently generated structures. The changes in free energy are detailed in Table 4.

**Table 4:** Energy scores resulting from each protein structure. Listed are the changes in free energy for each protein-sorbitol complex with corresponding descriptions of the complex and/or Python code run on the complex

Complex	Energy Score	Details
New Protein	-140.67 (protein only: -171.51)	Protein-sorbitol complex
New Protein After RepeatMover	-143.10	Protein-sorbitol complex with iteration via RepeatMover code with variables kT = 1 and n = 20
New Protein After MinMover	-214.97	Protein-sorbitol complex with backbone angles adjusted via MinMover code
Mutated Protein (All Binding Pocket Residues) After PackMover	-178.03	Mutated protein-sorbitol complex via PackMover (simulated annealing and Metropolis/Monte Carlo to find best residues and rotamers) for all 18 residues within four Angstroms of the binding pocket
Mutated Protein (Selected Binding Pocket Residues) After PackMover	-224.80 (protein only: -228.27)	Mutated protein-sorbitol complex via PackMover (simulated annealing and Metropolis/Monte Carlo to find best residues and rotamers) for a selected 5 residues within four Angstroms of the binding pocket
New Protein After FastRelax	-780.43	Protein-sorbitol complex with backbone angles adjusted and side chains repacked via FastRelax code

### Thermodynamic energy cycle

Given the thermodynamic equation below, which estimates binding free energy

$${\varDelta G}_{bind}={G}_{C}-({G}_{P}+{G}_{L})$$

and

$$\varDelta {G}_{bind}^{*}={G}_{C*}-({G}_{P*}+{G}_{L})$$

we derive the following equation

$$\begin{array}{c}\Delta \Delta {G_{bind}} = \Delta G_{bind}^* - \Delta {G_{bind}}\\= {G_{C*}} - {G_{P*}} - {G_C} + {G_P}\end{array}$$

which results in a unitless number that describes the thermodynamic cycle of energy for our protein. Plugging in numbers, for our new protein and protein-sorbitol complex versus our selectively mutated protein and protein-sorbitol complex with five residues mutated, we find that

$$\varDelta {\varDelta G}_{bind}={G}_{C*}-{G}_{P*}-{G}_{C}+{G}_{P}$$

$$\begin{array}{l}= \left( { - 224.80} \right) - \left( { - 228.27} \right) - \left( { - 140.67} \right)\\+ \left( { - 171.51} \right) = - 27.37\end{array}$$

As$\varDelta {\varDelta G}_{bind}<0$, the mutated version of the complex is thermodynamically favorable, and thus we can conclude that the selective mutations allowed for superior protein-ligand binding compared to the starting protein. It is important to note, however, that stabilizing the protein without the ligand in place such as with MinMover prior to mutation would not optimize for the protein when it is bound to the ligand. Thus, it is important that in the above calculation we used the energy score of the new protein prior to any minimization code (MinMover, Fast Relax, etc.) being run (Fig. 5).

## Conclusion

By applying PyRossetta’s suite of energy minimization algorithms to our protein of interest, we identified a protein with improved stability for binding sorbitol as compared to its original form. These algorithms stochastically induce amino acid residue mutations and conformational changes to identify proteins that are more energetically favorable. One reason our engineered protein is energetically favorable is due to the increased polar bonds. While other combinatorial changes can be attributed to the improved binding energy, these factors cannot be directly identified as they are a result of the algorithms in the PyRosetta suite.

Often, aldohexoses such as D-glucose, are found in a ringed form instead of a linear form. Given this information, there is only a small number of aldohexoses that are naturally occurring in their linear form that could compete with sorbitol for the binding spot of an engineered protein tuned for sorbitol selectivity [22–24]. Furthermore, as a therapy targeting diseases with sorbitol excess, we can ensure that the predominant substrate for binding the engineered protein will be sorbitol, rather than an alternative aldohexose that would possibly be found in the same region in relatively smaller amounts [22]. We can therefore ensure high selectivity for sorbitol.

Based on the structure of sorbitol, we hypothesized that by changing non-charged side chains to charged side chains within the binding pocket, the resulting conformational changes could allow the binding pocket to form new polar bonds with the hydroxyl groups in sorbitol and thus achieve greater stabilization [25]. As hydroxyl groups contain an oxygen with a partial negative charge and a hydrogen with partial positive charge, we can predict a change for a particular binding pocket amino acid side chain given a different one may be more energetically favorable. Interestingly, no mutative changes in the binding pocket itself were found to be more energetically favorable (Table 2).

Of the five selective changes within 4 Angstroms of the binding pocket (Table 3), the one residue that was hydrophobic changed to positive (PHE to LYS) may align with the hypothesis that polar bonds are more energetically favorable. For the two residues that were negative changed to neutral (ASP to PRO, ASP to TYR), and the remaining mutation (CYS to ILE), these may have occurred to further stabilize the structure and accommodate other changes, though indirectly. The last case (VAL to VAL) signified no mutation, which suggests that this residue, regardless of location, does not participate in the stabilization. Overall, the selective mutations were consistent with our predictions that adding more polar bonds to the binding pocket improves binding with sorbitol and improves stability of the protein. After PackMover mutations and FastRelax minimization, there were 10 polar bonds present amongst the 10 residues versus 7 polar bonds at the starting point. The lack of new polar bonds in the mutated protein and the gain of polar bonds in the FastRelax protein suggest that of the two strategies to decrease free energy through structural minimization of the protein-ligand complex, mutating proteins is less effective than rotational conformation. The results of the free energy changes in Table 4 corroborated the assumption that each strategy yielded protein-sorbitol complexes that were more energetically favorable than the original.

The mutations and conformations discussed above may be beneficial as they stabilize the binding of the ATP transporter SBP with sorbitol. This selectively mutated protein isolated from *Agrobacterium vitis* is a potential tool for sorbitol sequestration which could be used as a molecular sponge to preferentially bind and remove sorbitol from tissue, and thus potentially be utilized as a treatment for sorbitol dehydrogenase deficiency. Future development of this biologic and subsequent in vitro and in vivo experimentation can be utilized to study its efficacy.

### Limitations

The theoretical mutations developed in this work were developed by utilizing a Python modeling interface based on an underlying stochastic algorithm. It can be postulated that the various components of the code could be rerun and return further novel potential mutations with superior or inferior binding outcomes. Furthermore, while we chose to selectively administer mutations to the binding pocket, an automatic mode for determining which residues should be changed could provide for different outcomes. Future studies which incorporate in vitro and subsequently in vivo experimentation are necessary for effect confirmation. General limitations regarding biologics including side effects should be considered and further explored in future studies.

## Electronic supplementary material

Below is the link to the electronic supplementary material: https://github.com/ShonitNS/Sorbitol).

Supplementary Material 1

## Data Availability

1. ‘Chemical Components in the PDB: SOR,’ Protein Data Bank in Europe. https://www.ebi.ac.uk/pdbe-srv/pdbechem/chemicalCompound/show/SOR, October 2021. 2. ‘Chemical Components in the PDB: X9X,’ Protein Data Bank in Europe. https://www.ebi.ac.uk/pdbe-srv/pdbechem/chemicalCompound/show/X9X, October 2021. 3. ‘OpenBabel.’ ChemInfo. http://www.cheminfo.org/Chemistry/Cheminformatics/FormatConverter/index.html, October 2021. 4. PyMOL by Schrödinger. https://pymol.org/2/, October 2021. 5. ‘Proteomes – Argobacterium vitis (strain S4 / ATCC BAA-846) (Rhizobium vitis (strain S4)),’ UniProt. https://www.uniprot.org/proteomes/UP000001596, October 2021. 6. ‘SOR.’ Research Collaboratory for Structural Bioinformatics Protein Data Bank. https://www.rcsb.org/ligand/SOR, October 2021. 7. Research Collaboratory for Structural Bioinformatics Protein Data Bank. https://www.rcsb.org/, October 2021. 8. X9X.’ Research Collaboratory for Structural Bioinformatics Protein Data Bank. https://www.rcsb.org/ligand/X9X, October 2021. 9. ‘4WT7.’ Research Collaboratory for Structural Bioinformatics Protein Data Bank. https://www.rcsb.org/structure/4WT7, October 2021.

## Abbreviations

ABC: Adenosine Triphosphate-Binding Protein Cassette
Allitol: D-allitol
ALLAA: All amino acids, all repackaging
NATRO: No mutation, no repackaging
PDB: Protein Data Bank
RCSB: Research Collaboratory for Structural Bioinformatics Protein Data Bank
SBP: Solute Binding Protein

## References

[1] Brownlee M (2005). The pathobiology of diabetic complications: a unifying mechanism. Diabetes.

[2] Bhagavan NV, Bhagavan NV (2002). CHAPTER 9 - simple carbohydrates. Medical biochemistry (Fourth Edition).

[3] Leroith D, Taylor MD, Olefsky SI JM, editors. Diabetes Mellitus: A Fundamental and Clinical Text. 3rd edition. Philadelphia: Lippincott Williams & Wilkins; 2003.

[4] Safi SZ, Qvist R, Kumar S, Batumalaie K, Ismail ISB (2014). Molecular mechanisms of diabetic retinopathy, general preventive strategies, and novel therapeutic targets. Biomed Res Int.

[5] Jedziniak JA, Chylack LT, Cheng HM, Gillis MK, Kalustian AA, Tung WH (1981). The sorbitol pathway in the human lens: aldose reductase and polyol dehydrogenase. Investig Ophthalmol Vis Sci.

[6] Burg MB, Kador PF (1988). Sorbitol, osmoregulation, and the complications of diabetes. J Clin Invest.

[7] Hodgson N, Wu F, Zhu J, Wang W, Ferreyra H, Zhang K (2016). Economic and Quality of Life benefits of Anti-VEGF therapy. Mol Pharm.

[8] Cortese A, Zhu Y, Rebelo AP, Negri S, Courel S, Abreu L (2020). Biallelic mutations in SORD cause a common and potentially treatable hereditary neuropathy with implications for diabetes. Nat Genet.

[9] Vinson JA (2006). Oxidative stress in cataracts. Pathophysiology.

[10] Hashim Z, Zarina S (2012). Osmotic stress induced oxidative damage: possible mechanism of cataract formation in diabetes. J Diabetes Complicat.

[11] Li C, Zhang R, Wang J, Wilson LM, Yan Y (2020). Protein engineering for improving and diversifying natural products biosynthesis. Trends Biotechnol.

[12] Wu Z, Kan SBJ, Lewis RD, Wittmann BJ, Arnold FH (2019). Machine learning-assisted directed protein evolution with combinatorial libraries. Proc Natl Acad Sci.

[13] Rodriguez-Salazar. L(1), Guevara-Pulido J(2), Cifuentes A(2). In silico design of a peptide receptor for dopamine recognition. Molecules. 2020;25. 10.3390/molecules25235509 PMC7727804 33255517

[14] Hashemi ZS, Zarei M, Fath MK, Ganji M, Farahani MS, Afsharnouri F et al. In silico approaches for the design and optimization of interfering peptides against protein–protein interactions. Front Mol Biosci. 2021;8. 10.3389/fmolb.2021.669431 PMC8113820 33996914

[15] Adnan M, Khan S, Patel M, Al-Shammari E, Ashankyty IMA (2013). Agrobacterium Reviews and Research in Medical Microbiology.

[16] Yadava U, Vetting MW, Al Obaidi N, Carter MS, Gerlt JA, Almo SC (2016). Structure of an ABC transporter solute-binding protein specific for the amino sugars glucosamine and galactosamine. Acta Crystallogr F Struct Biol Commun.

[17] Vetting MW, Al Obaidi N, Toro R, Morisco LL, Benach J, Wasserman SR et al. Crystal structure of an ABC transporter solute binding protein (IPR025997) from Agrobacterium vitis (Avi_5165, Target EFI-511223) with bound allitol. 2014. https://www.rcsb.org/structure/4wt7. Accessed 25 Nov 2022.

[18] Darsow G. Process for the preparation of epimer-free sugar alcohols from the group consisting of xylitol, sorbitol (D-glucitol), and 4-O-α-D-glucopyranosyl-D-sorbitol. 1992.

[19] Azarnia N, Jeffrey GA, Shen MS (1972). The crystal structures of allitol and d-iditol. Acta Cryst B.

[20] OPENBABEL - Chemical file format converter. http://www.cheminfo.org/Chemistry/Cheminformatics/FormatConverter/index.html. Accessed 25 Nov 2022.

[21] PyMOL | pymol.org. https://pymol.org/2/. Accessed 25 Nov 2022.

[22] El-Kabbani O, Darmanin C, Chung RP-T (2004). Sorbitol dehydrogenase: structure, function and ligand design. Curr Med Chem.

[23] Chukwuma CI, Islam MdS (2017). Sorbitol increases muscle glucose uptake ex vivo and inhibits intestinal glucose absorption ex vivo and in normal and type 2 diabetic rats. Appl Physiol Nutr Metab.

[24] Adcock LH, Gray CH (1957). The metabolism of sorbitol in the human subject. Biochem J.

[25] Ng PC, Henikoff S (2006). Predicting the effects of amino acid substitutions on protein function. Annu Rev Genomics Hum Genet.

