# Supplementary material for: In-silico molecular designs to treat neurologic and ophthalmologic diseases caused by sorbitol excess: engineering the Agrobacterium vitis protein
Source: BMC Res Notes. 2023 Jul 3;16:129. doi: 10.1186/s13104-023-06367-2 (PMC10318746; doi:10.1186/s13104-023-06367-2)
Supplement: Supplementary file 1 — Supplementary Material 1 [file 13104_2023_6367_MOESM1_ESM.docx]

**In-silico molecular designs to treat neurologic and ophthalmologic diseases caused by sorbitol excess: engineering the *Agrobacterium vitis* protein**

Shonit Nair Sharma^1-3^, Ashkhan Hojati^3^, Rahul S. Yerrabelli^3^, Bhargavee Gnanasambandam^3*^, Joshua Brozek^4^

^1^ Department of Biological Engineering, Massachusetts Institute of Technology, Cambridge, MA 02139, USA

^2^ Brigham and Women’s Hospital, Harvard Medical School, Boston, MA 02115, USA

^3^ Carle Illinois College of Medicine, University of Illinois at Urbana-Champaign, Champaign, IL 61820, USA

^4^ Carle Foundation Hospital, Urbana, IL 61801, USA

*Corresponding Author: bg19@illinois.edu

Supplemental File

Author contacts:
SNS Email: shonit@mit.edu ORCID: 0000-0003-1016-647X

AH Email: ahojati2@illinois.edu ORCID: 0000-0001-5883-3257
RSY Email: rsy2@illinois.edu ORCID: 0000-0002-7670-9601

BG Email: bg19@illinois.edu ORCID: 0000-0001-5716-1620

**Appendix**

Full Python code: Sorbitol.ipynb

<http://nbviewer.jupyter.org>

Energy score for ligand-receptor:

------------------------------------------------------------

Scores Weight Raw Score Wghtd.Score

------------------------------------------------------------

fa_atr 1.000 -1779.287 -1779.287

fa_rep 0.550 325.328 178.931

fa_sol 1.000 1104.404 1104.404

fa_intra_rep 0.005 712.868 3.564

fa_intra_sol_xover4 1.000 58.952 58.952

lk_ball_wtd 1.000 -36.986 -36.986

fa_elec 1.000 -388.394 -388.394

pro_close 1.250 84.003 105.004

hbond_sr_bb 1.000 -117.706 -117.706

hbond_lr_bb 1.000 -59.811 -59.811

hbond_bb_sc 1.000 -29.524 -29.524

hbond_sc 1.000 -34.027 -34.027

dslf_fa13 1.250 0.000 0.000

omega 0.400 230.291 92.116

fa_dun 0.700 871.389 609.972

p_aa_pp 0.600 -57.568 -34.541

yhh_planarity 0.625 5.217 3.261

ref 1.000 122.013 122.013

rama_prepro 0.450 136.433 61.395

---------------------------------------------------

Total weighted score: -140.665

RepeatMover (kT = 1, n = 20):

original: -140.66486234243547

new: -143.10411740292957

PackMover after selecting all residues in the binding pocket to mutate to best residue/rotamer:

core.pack.task: Packer task: initialize from command line()

core.pack.pack_rotamers: built 5182 rotamers at 18 positions.

core.pack.interaction_graph.interaction_graph_factory: Instantiating PDInteractionGraph

Original Score: -140.66486234243547

Score after mutating residues: -178.02964183197446

MinMover to find a local energy minimum:

Original Energy: -140.66

Energy after min_mover: -214.97

PackMover after editing the resfile to change 5 residues and then string together multiple movers into a sequence:

core.pack.pack_rotamers: built 497 rotamers at 5 positions.

core.pack.interaction_graph.interaction_graph_factory: Instantiating PDInteractionGraph

Score: -222.46479950286252

core.pack.pack_rotamers: built 509 rotamers at 5 positions.

core.pack.interaction_graph.interaction_graph_factory: Instantiating PDInteractionGraph

Score: -224.3106954207604

core.pack.pack_rotamers: built 508 rotamers at 5 positions.

core.pack.interaction_graph.interaction_graph_factory: Instantiating PDInteractionGraph

Score: -224.75331083117842

core.pack.pack_rotamers: built 510 rotamers at 5 positions.

core.pack.interaction_graph.interaction_graph_factory: Instantiating PDInteractionGraph

Score: -224.785228591257

core.pack.pack_rotamers: built 509 rotamers at 5 positions.

core.pack.interaction_graph.interaction_graph_factory: Instantiating PDInteractionGraph

Score: -224.7899859391805

core.pack.pack_rotamers: built 510 rotamers at 5 positions.

core.pack.interaction_graph.interaction_graph_factory: Instantiating PDInteractionGraph

Score: -224.79511872238396

core.pack.pack_rotamers: built 509 rotamers at 5 positions.

core.pack.interaction_graph.interaction_graph_factory: Instantiating PDInteractionGraph

Score: -224.7967514068797

core.pack.pack_rotamers: built 510 rotamers at 5 positions.

core.pack.interaction_graph.interaction_graph_factory: Instantiating PDInteractionGraph

Score: -224.797721058859

core.pack.pack_rotamers: built 510 rotamers at 5 positions.

core.pack.interaction_graph.interaction_graph_factory: Instantiating PDInteractionGraph

Score: -224.79863793061156

core.pack.pack_rotamers: built 510 rotamers at 5 positions.

core.pack.interaction_graph.interaction_graph_factory: Instantiating PDInteractionGraph

Score: -224.79952257666133

core.pack.pack_rotamers: built 510 rotamers at 5 positions.

core.pack.interaction_graph.interaction_graph_factory: Instantiating PDInteractionGraph

Score: -224.80038170950328

LYS

PRO

TYR

VAL

ILE

FastRelax:

protocols.relax.RelaxScriptManager: Reading relax scripts list from database.

core.scoring.ScoreFunctionFactory: SCOREFUNCTION: ref2015

protocols.relax.RelaxScriptManager: Looking for MonomerRelax2019.txt

protocols.relax.RelaxScriptManager: ================== Reading script file: /usr/local/lib/python3.7/dist-packages/pyrosetta-2021.35+release.4431687773b-py3.7-linux-x86_64.egg/pyrosetta/database/sampling/relax_scripts/MonomerRelax2019.txt ==================

protocols.relax.RelaxScriptManager: repeat %%nrepeats%%

protocols.relax.RelaxScriptManager: coord_cst_weight 1.0

protocols.relax.RelaxScriptManager: scale:fa_rep 0.040

protocols.relax.RelaxScriptManager: repack

protocols.relax.RelaxScriptManager: scale:fa_rep 0.051

protocols.relax.RelaxScriptManager: min 0.01

protocols.relax.RelaxScriptManager: coord_cst_weight 0.5

protocols.relax.RelaxScriptManager: scale:fa_rep 0.265

protocols.relax.RelaxScriptManager: repack

protocols.relax.RelaxScriptManager: scale:fa_rep 0.280

protocols.relax.RelaxScriptManager: min 0.01

protocols.relax.RelaxScriptManager: coord_cst_weight 0.0

protocols.relax.RelaxScriptManager: scale:fa_rep 0.559

protocols.relax.RelaxScriptManager: repack

protocols.relax.RelaxScriptManager: scale:fa_rep 0.581

protocols.relax.RelaxScriptManager: min 0.01

protocols.relax.RelaxScriptManager: coord_cst_weight 0.0

protocols.relax.RelaxScriptManager: scale:fa_rep 1

protocols.relax.RelaxScriptManager: repack

protocols.relax.RelaxScriptManager: min 0.00001

protocols.relax.RelaxScriptManager: accept_to_best

protocols.relax.RelaxScriptManager: endrepeat

protocols.relax.FastRelax: CMD: repeat -140.665 0 0 0.55

protocols.relax.FastRelax: CMD: coord_cst_weight -140.665 0 0 0.55

protocols.relax.FastRelax: CMD: scale:fa_rep -312.438 0 0 0.022

core.pack.task: Packer task: initialize from command line()

core.pack.rotamer_set.RotamerSet_: Using simple Rotamer generation logic for LG1

core.pack.pack_rotamers: built 9197 rotamers at 295 positions.

core.pack.interaction_graph.interaction_graph_factory: Instantiating DensePDInteractionGraph

protocols.relax.FastRelax: CMD: repack -790.102 0 0 0.022

protocols.relax.FastRelax: CMD: scale:fa_rep -779.923 0 0 0.02805

protocols.relax.FastRelax: CMD: min -1301.28 1.09235 1.09235 0.02805

protocols.relax.FastRelax: CMD: coord_cst_weight -1301.28 1.09235 1.09235 0.02805

protocols.relax.FastRelax: CMD: scale:fa_rep -693.655 1.09235 1.09235 0.14575

core.pack.task: Packer task: initialize from command line()

core.pack.rotamer_set.RotamerSet_: Using simple Rotamer generation logic for LG1

core.pack.pack_rotamers: built 8635 rotamers at 295 positions.

core.pack.interaction_graph.interaction_graph_factory: Instantiating DensePDInteractionGraph

protocols.relax.FastRelax: CMD: repack -724.717 1.09235 1.09235 0.14575

protocols.relax.FastRelax: CMD: scale:fa_rep -686.558 1.09235 1.09235 0.154

protocols.relax.FastRelax: CMD: min -1042.58 0.733614 0.733614 0.154

protocols.relax.FastRelax: CMD: coord_cst_weight -1042.58 0.733614 0.733614 0.154

protocols.relax.FastRelax: CMD: scale:fa_rep -855.442 0.733614 0.733614 0.30745

core.pack.task: Packer task: initialize from command line()

core.pack.rotamer_set.RotamerSet_: Using simple Rotamer generation logic for LG1

core.pack.pack_rotamers: built 8311 rotamers at 295 positions.

core.pack.interaction_graph.interaction_graph_factory: Instantiating DensePDInteractionGraph

protocols.relax.FastRelax: CMD: repack -857.367 0.733614 0.733614 0.30745

protocols.relax.FastRelax: CMD: scale:fa_rep -842.815 0.733614 0.733614 0.31955

protocols.relax.FastRelax: CMD: min -887.221 0.613766 0.613766 0.31955

protocols.relax.FastRelax: CMD: coord_cst_weight -887.221 0.613766 0.613766 0.31955

protocols.relax.FastRelax: CMD: scale:fa_rep -706.797 0.613766 0.613766 0.55

core.pack.task: Packer task: initialize from command line()

core.pack.rotamer_set.RotamerSet_: Using simple Rotamer generation logic for LG1

core.pack.pack_rotamers: built 8110 rotamers at 295 positions.

core.pack.interaction_graph.interaction_graph_factory: Instantiating DensePDInteractionGraph

protocols.relax.FastRelax: CMD: repack -707.358 0.613766 0.613766 0.55

protocols.relax.FastRelax: CMD: min -767.944 0.511685 0.511685 0.55

protocols.relax.FastRelax: MRP: 0 -767.944 -767.944 0.511685 0.511685

protocols.relax.FastRelax: CMD: accept_to_best -767.944 0.511685 0.511685 0.55

protocols.relax.FastRelax: CMD: endrepeat -767.944 0.511685 0.511685 0.55

protocols.relax.FastRelax: CMD: coord_cst_weight -767.944 0.511685 0.511685 0.55

protocols.relax.FastRelax: CMD: scale:fa_rep -1029.55 0.511685 0.511685 0.022

core.pack.task: Packer task: initialize from command line()

core.pack.rotamer_set.RotamerSet_: Using simple Rotamer generation logic for LG1

core.pack.pack_rotamers: built 9444 rotamers at 295 positions.

core.pack.interaction_graph.interaction_graph_factory: Instantiating DensePDInteractionGraph

protocols.relax.FastRelax: CMD: repack -1052.83 0.511685 0.511685 0.022

protocols.relax.FastRelax: CMD: scale:fa_rep -1047.25 0.511685 0.511685 0.02805

protocols.relax.FastRelax: CMD: min -1328.24 1.16135 1.16135 0.02805

protocols.relax.FastRelax: CMD: coord_cst_weight -1328.24 1.16135 1.16135 0.02805

protocols.relax.FastRelax: CMD: scale:fa_rep -712.071 1.16135 1.16135 0.14575

core.pack.task: Packer task: initialize from command line()

core.pack.rotamer_set.RotamerSet_: Using simple Rotamer generation logic for LG1

core.pack.pack_rotamers: built 8624 rotamers at 295 positions.

core.pack.interaction_graph.interaction_graph_factory: Instantiating DensePDInteractionGraph

protocols.relax.FastRelax: CMD: repack -743.202 1.16135 1.16135 0.14575

protocols.relax.FastRelax: CMD: scale:fa_rep -704.26 1.16135 1.16135 0.154

protocols.relax.FastRelax: CMD: min -1054.63 0.755542 0.755542 0.154

protocols.relax.FastRelax: CMD: coord_cst_weight -1054.63 0.755542 0.755542 0.154

protocols.relax.FastRelax: CMD: scale:fa_rep -865.697 0.755542 0.755542 0.30745

core.pack.task: Packer task: initialize from command line()

core.pack.rotamer_set.RotamerSet_: Using simple Rotamer generation logic for LG1

core.pack.pack_rotamers: built 8245 rotamers at 295 positions.

core.pack.interaction_graph.interaction_graph_factory: Instantiating DensePDInteractionGraph

protocols.relax.FastRelax: CMD: repack -867.6 0.755542 0.755542 0.30745

protocols.relax.FastRelax: CMD: scale:fa_rep -852.926 0.755542 0.755542 0.31955

protocols.relax.FastRelax: CMD: min -901.168 0.643815 0.643815 0.31955

protocols.relax.FastRelax: CMD: coord_cst_weight -901.168 0.643815 0.643815 0.31955

protocols.relax.FastRelax: CMD: scale:fa_rep -722.322 0.643815 0.643815 0.55

core.pack.task: Packer task: initialize from command line()

core.pack.rotamer_set.RotamerSet_: Using simple Rotamer generation logic for LG1

core.pack.pack_rotamers: built 8046 rotamers at 295 positions.

core.pack.interaction_graph.interaction_graph_factory: Instantiating DensePDInteractionGraph

protocols.relax.FastRelax: CMD: repack -723.537 0.643815 0.643815 0.55

protocols.relax.FastRelax: CMD: min -780.433 0.619378 0.619378 0.55

protocols.relax.FastRelax: MRP: 1 -780.433 -780.433 0.619378 0.619378

protocols.relax.FastRelax: CMD: accept_to_best -780.433 0.619378 0.619378 0.55

protocols.relax.FastRelax: CMD: endrepeat -780.433 0.619378 0.619378 0.55

protocols.relax.FastRelax: CMD: coord_cst_weight -780.433 0.619378 0.619378 0.55

protocols.relax.FastRelax: CMD: scale:fa_rep -1045.74 0.619378 0.619378 0.022

core.pack.task: Packer task: initialize from command line()

core.pack.rotamer_set.RotamerSet_: Using simple Rotamer generation logic for LG1

core.pack.pack_rotamers: built 9270 rotamers at 295 positions.

core.pack.interaction_graph.interaction_graph_factory: Instantiating DensePDInteractionGraph

protocols.relax.FastRelax: CMD: repack -1065.66 0.619378 0.619378 0.022

protocols.relax.FastRelax: CMD: scale:fa_rep -1060.26 0.619378 0.619378 0.02805

protocols.relax.FastRelax: CMD: min -1331.26 1.17739 1.17739 0.02805

protocols.relax.FastRelax: CMD: coord_cst_weight -1331.26 1.17739 1.17739 0.02805

protocols.relax.FastRelax: CMD: scale:fa_rep -739.142 1.17739 1.17739 0.14575

core.pack.task: Packer task: initialize from command line()

core.pack.rotamer_set.RotamerSet_: Using simple Rotamer generation logic for LG1

core.pack.pack_rotamers: built 8581 rotamers at 295 positions.

core.pack.interaction_graph.interaction_graph_factory: Instantiating DensePDInteractionGraph

protocols.relax.FastRelax: CMD: repack -769.685 1.17739 1.17739 0.14575

protocols.relax.FastRelax: CMD: scale:fa_rep -732.035 1.17739 1.17739 0.154

protocols.relax.FastRelax: CMD: min -1062.04 0.765567 0.765567 0.154

protocols.relax.FastRelax: CMD: coord_cst_weight -1062.04 0.765567 0.765567 0.154

protocols.relax.FastRelax: CMD: scale:fa_rep -874.978 0.765567 0.765567 0.30745

core.pack.task: Packer task: initialize from command line()

core.pack.rotamer_set.RotamerSet_: Using simple Rotamer generation logic for LG1

core.pack.pack_rotamers: built 8261 rotamers at 295 positions.

core.pack.interaction_graph.interaction_graph_factory: Instantiating DensePDInteractionGraph

protocols.relax.FastRelax: CMD: repack -875.512 0.765567 0.765567 0.30745

protocols.relax.FastRelax: CMD: scale:fa_rep -861.015 0.765567 0.765567 0.31955

protocols.relax.FastRelax: CMD: min -906.554 0.66232 0.66232 0.31955

protocols.relax.FastRelax: CMD: coord_cst_weight -906.554 0.66232 0.66232 0.31955

protocols.relax.FastRelax: CMD: scale:fa_rep -731.435 0.66232 0.66232 0.55

core.pack.task: Packer task: initialize from command line()

core.pack.rotamer_set.RotamerSet_: Using simple Rotamer generation logic for LG1

core.pack.pack_rotamers: built 7977 rotamers at 295 positions.

core.pack.interaction_graph.interaction_graph_factory: Instantiating DensePDInteractionGraph

protocols.relax.FastRelax: CMD: repack -731.9 0.66232 0.66232 0.55

protocols.relax.FastRelax: CMD: min -780.342 0.611398 0.611398 0.55

protocols.relax.FastRelax: MRP: 2 -780.342 -780.433 0.619378 0.619378

protocols.relax.FastRelax: CMD: accept_to_best -780.342 0.611398 0.611398 0.55

protocols.relax.FastRelax: CMD: endrepeat -780.342 0.611398 0.611398 0.55

protocols.relax.FastRelax: CMD: coord_cst_weight -780.342 0.611398 0.611398 0.55

protocols.relax.FastRelax: CMD: scale:fa_rep -1046.5 0.611398 0.611398 0.022

core.pack.task: Packer task: initialize from command line()

core.pack.rotamer_set.RotamerSet_: Using simple Rotamer generation logic for LG1

core.pack.pack_rotamers: built 9255 rotamers at 295 positions.

core.pack.interaction_graph.interaction_graph_factory: Instantiating DensePDInteractionGraph

protocols.relax.FastRelax: CMD: repack -1065.27 0.611398 0.611398 0.022

protocols.relax.FastRelax: CMD: scale:fa_rep -1059.39 0.611398 0.611398 0.02805

protocols.relax.FastRelax: CMD: min -1334.43 1.20093 1.20093 0.02805

protocols.relax.FastRelax: CMD: coord_cst_weight -1334.43 1.20093 1.20093 0.02805

protocols.relax.FastRelax: CMD: scale:fa_rep -723.142 1.20093 1.20093 0.14575

core.pack.task: Packer task: initialize from command line()

core.pack.rotamer_set.RotamerSet_: Using simple Rotamer generation logic for LG1

core.pack.pack_rotamers: built 8651 rotamers at 295 positions.

core.pack.interaction_graph.interaction_graph_factory: Instantiating DensePDInteractionGraph

protocols.relax.FastRelax: CMD: repack -738.49 1.20093 1.20093 0.14575

protocols.relax.FastRelax: CMD: scale:fa_rep -699.873 1.20093 1.20093 0.154

protocols.relax.FastRelax: CMD: min -1060.91 0.807136 0.807136 0.154

protocols.relax.FastRelax: CMD: coord_cst_weight -1060.91 0.807136 0.807136 0.154

protocols.relax.FastRelax: CMD: scale:fa_rep -872.561 0.807136 0.807136 0.30745

core.pack.task: Packer task: initialize from command line()

core.pack.rotamer_set.RotamerSet_: Using simple Rotamer generation logic for LG1

core.pack.pack_rotamers: built 8387 rotamers at 295 positions.

core.pack.interaction_graph.interaction_graph_factory: Instantiating DensePDInteractionGraph

protocols.relax.FastRelax: CMD: repack -873.153 0.807136 0.807136 0.30745

protocols.relax.FastRelax: CMD: scale:fa_rep -858.554 0.807136 0.807136 0.31955

protocols.relax.FastRelax: CMD: min -905.129 0.70271 0.70271 0.31955

protocols.relax.FastRelax: CMD: coord_cst_weight -905.129 0.70271 0.70271 0.31955

protocols.relax.FastRelax: CMD: scale:fa_rep -727.984 0.70271 0.70271 0.55

core.pack.task: Packer task: initialize from command line()

core.pack.rotamer_set.RotamerSet_: Using simple Rotamer generation logic for LG1

core.pack.pack_rotamers: built 8030 rotamers at 295 positions.

core.pack.interaction_graph.interaction_graph_factory: Instantiating DensePDInteractionGraph

protocols.relax.FastRelax: CMD: repack -728.377 0.70271 0.70271 0.55

protocols.relax.FastRelax: CMD: min -777.433 0.652461 0.652461 0.55

protocols.relax.FastRelax: MRP: 3 -777.433 -780.433 0.619378 0.619378

protocols.relax.FastRelax: CMD: accept_to_best -777.433 0.652461 0.652461 0.55

protocols.relax.FastRelax: CMD: endrepeat -777.433 0.652461 0.652461 0.55

protocols.relax.FastRelax: CMD: coord_cst_weight -777.433 0.652461 0.652461 0.55

protocols.relax.FastRelax: CMD: scale:fa_rep -1047.72 0.652461 0.652461 0.022

core.pack.task: Packer task: initialize from command line()

core.pack.rotamer_set.RotamerSet_: Using simple Rotamer generation logic for LG1

core.pack.pack_rotamers: built 9441 rotamers at 295 positions.

core.pack.interaction_graph.interaction_graph_factory: Instantiating DensePDInteractionGraph

protocols.relax.FastRelax: CMD: repack -1068.38 0.652461 0.652461 0.022

protocols.relax.FastRelax: CMD: scale:fa_rep -1063.09 0.652461 0.652461 0.02805

protocols.relax.FastRelax: CMD: min -1337.32 1.23823 1.23823 0.02805

protocols.relax.FastRelax: CMD: coord_cst_weight -1337.32 1.23823 1.23823 0.02805

protocols.relax.FastRelax: CMD: scale:fa_rep -722.672 1.23823 1.23823 0.14575

core.pack.task: Packer task: initialize from command line()

core.pack.rotamer_set.RotamerSet_: Using simple Rotamer generation logic for LG1

core.pack.pack_rotamers: built 8628 rotamers at 295 positions.

core.pack.interaction_graph.interaction_graph_factory: Instantiating DensePDInteractionGraph

protocols.relax.FastRelax: CMD: repack -750.579 1.23823 1.23823 0.14575

protocols.relax.FastRelax: CMD: scale:fa_rep -711.633 1.23823 1.23823 0.154

protocols.relax.FastRelax: CMD: min -1063.57 0.836218 0.836218 0.154

protocols.relax.FastRelax: CMD: coord_cst_weight -1063.57 0.836218 0.836218 0.154

protocols.relax.FastRelax: CMD: scale:fa_rep -874.465 0.836218 0.836218 0.30745

core.pack.task: Packer task: initialize from command line()

core.pack.rotamer_set.RotamerSet_: Using simple Rotamer generation logic for LG1

core.pack.pack_rotamers: built 8291 rotamers at 295 positions.

core.pack.interaction_graph.interaction_graph_factory: Instantiating DensePDInteractionGraph

protocols.relax.FastRelax: CMD: repack -876.213 0.836218 0.836218 0.30745

protocols.relax.FastRelax: CMD: scale:fa_rep -861.588 0.836218 0.836218 0.31955

protocols.relax.FastRelax: CMD: min -908.057 0.699899 0.699899 0.31955

protocols.relax.FastRelax: CMD: coord_cst_weight -908.057 0.699899 0.699899 0.31955

protocols.relax.FastRelax: CMD: scale:fa_rep -732.451 0.699899 0.699899 0.55

core.pack.task: Packer task: initialize from command line()

core.pack.rotamer_set.RotamerSet_: Using simple Rotamer generation logic for LG1

core.pack.pack_rotamers: built 7951 rotamers at 295 positions.

core.pack.interaction_graph.interaction_graph_factory: Instantiating DensePDInteractionGraph

protocols.relax.FastRelax: CMD: repack -732.801 0.699899 0.699899 0.55

protocols.relax.FastRelax: CMD: min -778.085 0.641572 0.641572 0.55

protocols.relax.FastRelax: MRP: 4 -778.085 -780.433 0.619378 0.619378

protocols.relax.FastRelax: CMD: accept_to_best -778.085 0.641572 0.641572 0.55

protocols.relax.FastRelax: CMD: endrepeat -778.085 0.641572 0.641572 0.55

protocols::checkpoint: Deleting checkpoints of FastRelax

Original Energy: -140.66

Energy after fast relax: -780.43
